# Higher polygenic risk for melanoma is associated with improved survival in a high ultraviolet radiation setting

**DOI:** 10.1186/s12967-022-03613-2

**Published:** 2022-09-05

**Authors:** Mathias Seviiri, Richard A. Scolyer, D. Timothy Bishop, Julia A. Newton-Bishop, Mark M. Iles, Serigne N. Lo, Johnathan R. Stretch, Robyn P. M. Saw, Omgo E. Nieweg, Kerwin F. Shannon, Andrew J. Spillane, Scott D. Gordon, Catherine M. Olsen, David C. Whiteman, Maria Teresa Landi, John F. Thompson, Georgina V. Long, Stuart MacGregor, Matthew H. Law

**Affiliations:** 1 Statistical Genetics Lab, QIMR Berghofer Medical Research Institute, 300 Herston Road, Herston, QLD 4006 Australia; 2 School of Biomedical Sciences, Faculty of Health, Queensland University of Technology, Brisbane, QLD Australia; 3 Center for Genomics and Personalised Health, Queensland University of Technology, Brisbane, QLD Australia; 4 Melanoma Institute Australia, The University of Sydney, Sydney, NSW Australia; 5 Faculty of Medicine and Health, The University of Sydney, Sydney, NSW Australia; 6 Department of Tissue Oncology and Diagnostic Pathology, Royal Prince Alfred Hospital, Sydney, NSW Australia; 7 NSW Health Pathology, Sydney, NSW Australia; 8 Division of Haematology and Immunology, Leeds Institute of Medical Research at St James’, University of Leeds, Leeds, UK; 9 St James’s Institute of Medical Research, University of Leeds, Leeds, UK; 10 Leeds Institute of Data Analytics, University of Leeds, Leeds, UK; 11 Department of Melanoma and Surgical Oncology, Royal Prince Alfred Hospital, Camperdown, NSW Australia; 12 Sydney Head & Neck Cancer Institute, Chris O’Brien Lifehouse Cancer Center, Sydney, NSW Australia; 13 Department of Breast and Melanoma Surgery, Royal North Shore Hospital, Sydney, NSW Australia; 14 Genetic Epidemiology Lab, QIMR Berghofer Medical Research Institute, Brisbane, QLD Australia; 15 Cancer Control Group, QIMR Berghofer Medical Research Institute, Brisbane, QLD Australia; 16 Faculty of Medicine, University of Queensland, Brisbane, QLD Australia; 17 Division of Cancer Epidemiology and Genetics, National Cancer Institute, National Institutes of Health, Bethesda, MD USA; 18 Department of Medical Oncology, Mater Hospital, North Sydney, NSW Australia; 19 Department of Medical Oncology, Royal North Shore Hospital, St Leonards, NSW Australia

**Keywords:** Melanoma, Melanoma-specific survival, Polygenic risk score, Genome-wide association study, Skin cancer

## Abstract

**Background:**

The role of germline genetic factors in determining survival from cutaneous melanoma (CM) is not well understood.

**Objective:**

To perform a genome-wide association study (GWAS) meta-analysis of melanoma-specific survival (MSS), and test whether a CM-susceptibility polygenic risk score (PRS) is associated with MSS.

**Methods:**

We conducted two Cox proportional-hazard GWAS of MSS using data from the Melanoma Institute Australia, a high ultraviolet (UV) radiation setting (MIA; 5,762 patients with melanoma; 800 melanoma deaths) and UK Biobank (UKB: 5,220 patients with melanoma; 241 melanoma deaths), and combined them in a fixed-effects meta-analysis. Significant (P < 5 × 10–8) results were investigated in the Leeds Melanoma Cohort (LMC; 1,947 patients with melanoma; 370 melanoma deaths). We also developed a CM-susceptibility PRS using a large independent GWAS meta-analysis (23,913 cases, 342,870 controls). The PRS was tested for an association with MSS in the MIA and UKB cohorts.

**Results:**

Two loci were significantly associated with MSS in the meta-analysis of MIA and UKB with lead SNPs rs41309643 (G allele frequency 1.6%, HR = 2.09, 95%CI = 1.61–2.71, P = 2.08 × 10–8) on chromosome 1, and rs75682113 (C allele frequency 1.8%, HR = 2.38, 95%CI = 1.77–3.21, P = 1.07 × 10–8) on chromosome 7. While neither SNP replicated in the LMC, rs75682113 was significantly associated in the combined discovery and replication sets. After adjusting for age at diagnosis, sex and the first ten principal components, a one standard deviation increase in the CM-susceptibility PRS was associated with improved MSS in the discovery meta-analysis (HR = 0.88, 95% CI = 0.83–0.94, P = 6.93 × 10–5; I2 = 88%). However, this was only driven by the high UV setting cohort (MIA HR = 0.84, 95% CI = 0.78–0.90).

**Conclusion:**

We found two loci potentially associated with MSS. Increased genetic susceptibility to develop CM is associated with improved MSS in a high UV setting.

**Supplementary Information:**

The online version contains supplementary material available at 10.1186/s12967-022-03613-2.

## Introduction

Cutaneous melanoma (CM) is the third most common skin cancer and is responsible for over 1,300 deaths in Australia annually [1] and more than 7,000 deaths in the United States of America (USA) [2]. While survival rates have been improving since 2013, likely due to advances in immunotherapies and BRAF-targeted therapies, management of CM remains a major public health burden, with an annual cost of over AUD 200 million in Australia and USD 24 billion in the US [3, 4].

CM-susceptibility is driven by host factors including skin pigmentation and number of naevi, as well as environmental factors, most importantly exposure to ultraviolet radiation [5–9]. Germline genetic factors can influence the risk of developing CM through modification of these host risk factors, and other biological pathways; genome‐wide association studies (GWAS) have identified over 50 CM-susceptibility loci [10].

Although there are well known prognostic factors for melanoma-specific survival (MSS) including primary tumour thickness, ulceration, mitotic rate, melanoma type, anatomical site and the stage of the tumour at diagnosis [11, 12], the role of host genetic factors in MSS is not well understood. Death of a relative from CM is associated with poorer MSS, raising the possibility that germline genetic factors influence survival [13]. Higher naevus count has been associated with improved survival [14]. Naevus count is strongly influenced by germline genetics [15, 16], and is the strongest risk factor for the development of melanoma [17], suggesting germline genetic risk for CM may also impact survival. Telomere length is another biological pathway to high genetic CM-susceptibility [18] and may also influence MSS [19].

A powerful approach to test whether germline genetic risk for a given disease or trait (e.g. risk for CM) influences another trait (e.g. MSS) is to combine individual genetic effects in a polygenic risk score (PRS). Death from all causes has been associated with the joint effect of PRSs associated with risk of a range of diseases (e.g. coronary artery disease, pancreatic cancer, and lung cancer) or associated with mortality risk factors (e.g. cholesterol, sleep duration) [20], suggesting that germline risk for development of a disease can help predict outcomes. However, it is not known whether a genetic predisposition to CM influences melanoma outcomes.

To explore these two questions, we first aimed to identify germline genetic factors that influence MSS by performing a large-scale GWAS of MSS. Following this we assessed whether a PRS for CM-susceptibility (referred to as PRS_susceptibility) was associated with MSS.

## Methods and materials

### Genome-wide association studies of melanoma-specific survival

#### Discovery cohort 1: Melanoma Institute Australia

Samples for this cohort were derived from the Melanoma Institute Australia (MIA) Biospecimen Bank (protocol HREC/10/RPAH/530) and patient information from the MIA Research Database (protocol HREC/11/RPAH/444). With written, informed consent, patients with histo-pathological confirmed CM cases managed at MIA, Sydney, Australia were identified from this Biospecimen Bank and Database. Participants’ clinical and biospecimen data were captured and prospectively collected follow- up for outcomes including death due to melanoma. MIA study protocols were approved by the Sydney Local Health District Ethics Review Committee, Royal Prince Alfred Hospital, Camperdown, Australia. Participants were genotyped in phases using the Oncoarray in 2014 and 2016, and the Global Screening Array in 2018 (Illumina, San Diego).

Full details of the GWAS data cleaning quality control for both MIA datasets have been previously reported [10, 21]. Briefly, for Oncoarray genotyped samples, individuals were removed based on high genotype missingness (> 3%), extreme heterozygosity (± 0.05 from the mean), being related to other samples (identified by descent pihat > 0.15), or were more than 6 standard deviations (SDs) from the means of principal components (PCs) 1 and 2 of a European reference population [10]. In addition, single nucleotide polymorphisms (SNPs) were removed if they were missing more than 3% of their calls, had a minor allele frequency (MAF) < 0.01, or their Hardy–Weinberg equilibrium (HWE) P-value was less than 5 × 10^−10^ for patients with melanoma or less than 5 × 10^−4^ in CM-free individuals in Landi et al., [10]. Individuals genotyped on the Global Screening Array were removed due to high genotype missingness (> 5%), non-European ancestry or relatedness (as above), and SNPs were excluded due to a low MAF (< 0.01), high missingness (> 5%), HWE P < 1 × 10^–6^, or a low GenTrain score (< 0.6) [21]. The cleaned genotyped data were batched by their genotyping array (Oncoarray and Global Screening Array) and imputed to the Haplotype Reference Consortium (v1) panel using the University of Michigan imputation server [22].

For this study, the primary endpoint was death from melanoma which was ascertained through MIA clinical records and linkage to Australian Cancer Registries (including the New South Wales Cancer Registry), electoral rolls, and the Birth and Death Register. This analysis was restricted to 5,672 participants of European ancestry diagnosed with CM. For participants with multiple CM, the first primary CM was used to define the start point. MSS survival time (in years) was defined as the duration between the date of diagnosis of the (first) primary CM, and the date of death due to melanoma. Patients were censored on the last day of follow-up or when they died of non-melanoma causes.

#### Discovery cohort 2: UK Biobank

UK Biobank (UKB) is a large population-based cohort of approximately 500,000 adult participants (40–70 years at recruitment) recruited with informed consent from the United Kingdom between 2006 and 2010. Participants were followed up for disease outcomes including death from melanoma. Details on participant recruitment, phenotype measurement and genotyping have been published elsewhere [23, 24]. In brief, participants were genotyped using the UK Biobank Axiom Array and the UK BiLEVE Axiom Array (Affymetrix Inc, California, USA) and imputed using the Haplotype Reference Consortium and UK10K reference panels. The study was approved by the United Kingdom’s National North West Multi-Centre Research Ethics Committee. For this present study, we included 5,220 participants of European ancestry with histo-pathologically confirmed invasive CM based on the International Classification of Diseases (ICD) 10 (UKB data field 40,006) and 9 (data field 40,013) and ICD for Oncology, 3rd edition codes (data field 40,011) for melanoma. Participants were then filtered for missingness (< 3%), relatedness (identity by descent pihat < 0.2), and population ancestry outliers (from the European reference). The primary endpoint was MSS which was ascertained through linkage of the participant records with Cancer Registries, electoral rolls, and the Birth and Death Register in the UK.

### Replication cohort: Leeds Melanoma Cohort

The Leeds Melanoma Cohort (LMC) is a population-based cohort of 2,184 participants diagnosed with incident melanoma between September 2000 and December 2012 and residing in Yorkshire and the North of England [25]. Details on the recruitment, follow-up and phenotype/genotype data processing have been published previously [25, 26, 27]. In brief, for two periods (September 2000–December 2001, and July 2003 to December 2005) recruitment was restricted to patients with a primary tumour thickness of > 0.75 mm, while all patients with invasive melanoma were invited to participate between January 2002 and June 2003, and between January 2006–2012. Melanoma survival information was collected by direct communication with patients and their families, clinical records and from national registers.

Melanoma diagnoses were clinico-histopathologically confirmed through data linkage with the Northern and Yorkshire Cancer Registry and Information Service. Samples were genotyped using the Infinium HumanOmniExpressExome array (Illumina San Diego, CA, USA). After genetic quality control procedures (filtering for missingness, relatedness, and population outliers), this present study was restricted to 1,947 participants with genetic and phenotype data, and consent. Ethical approval for research involving the LMC was obtained from the Northern and Yorkshire Research Ethics Committee, and all participants provided written informed consent.

SNPs with MAF < 0.03, control Hardy–Weinberg equilibrium (HWE) P < 10^–4^ or missingness > 0.03 were excluded, as were any individuals with call rates < 0.97, identified as first degree relatives and/or European outliers by principal components analysis. Samples were imputed using the Haplotype Reference Consortium panel at the University of Michigan imputation server [22] and variants with an imputation quality score < 0.5 or MAF < 0.0001 were discarded.

### Statistical analysis: genome-wide association study of melanoma-specific survival

First, we conducted two GWAS of MSS in the MIA cohort (5762 patients with melanoma and 800 melanoma-specific deaths) and in UKB cohort (5220 melanoma patients and 241 melanoma-specific deaths). Using Cox proportional-hazard modelling, hazard ratios (HRs) were computed using PLINK 1.9 [28] and the R *survival* package [29]. In both the MIA and UKB analyses, we adjusted for age, sex and the first ten PCs; in the MIA cohort we also adjusted for genotyping batch. We defined survival time as the duration between the date of diagnosis of the (first) primary CM and the date of death due to melanoma, measured in years. Other participants were censored on the last day of follow-up or when they died of any other cause. Analysis was restricted to participants of European ancestry and SNPs with MAF > 0.5%, and an imputation quality score > 0.5.

Next, we conducted a meta-analysis for both GWAS (N = 10,982 and 1,041 melanoma deaths) using a fixed-effects inverse-variance weighted model in METAL [30]. In addition, measures of heterogeneity (such as I^2^) were computed. Lead genome-wide significant (P < 5 × 10^–8^) SNPs independent at linkage disequilibrium (LD) r^2^ < 0.1 were identified using FUMA v1.3.6a (https://fuma.ctglab.nl/) [31].

Lead SNPs were tested for replication in the LMC (N = 1,947 patients with melanoma and 370 melanomas-specific deaths). The replication p-value threshold was set to 0.05. Next, we conducted a fixed- and random- effects inverse-variance meta-analysis of the lead SNPs from all three sets (MIA, UKB and LMC) using METAL [30]. For the two lead SNPs the nearest gene, and any significant expression quantitative trait loci (eQTLs) were identified using FUMA v1.3.6a [31].

### Cutaneous melanoma polygenic risk score

#### Cutaneous melanoma risk discovery cohorts and GWAS meta-analysis

As the three MSS GWAS cohorts contributed to the discovery CM-susceptibility GWAS meta-analysis [10], and overlap between datasets used to generate, optimise or test PRS can lead to overfitting and other biases [32], we re-analysed the CM-susceptibility GWAS meta-analysis excluding the three MSS GWAS datasets. We further excluded the QSkin Sun and Health Study cohort to use as an independent data set to validate the generated PRSs. Details on recruitment, case definitions, genotyping, quality control, imputation approaches and ethical approvals for each cohort have been extensively described before [10]. The updated meta-analysis consisted of 23,913 cases, and 342,870 controls of European ancestry from Europe, Australia and the United States of America (USA) (Additional file 2: Table 1).

**Table 1:** Characteristics of Melanoma Institute Australia, UK Biobank and Leeds Melanoma Cohorts

Characteristic	MIA	UKB	LMC
Number	5762	5220	1947
Mean age at first diagnosis in years (SD)	60.1 (15.4)	56.78 (11.2)	55.05 (13.4)
Number of males (%)	3,478 (60.4)	2,231 (42.7)	839 (43.1)
Mean duration of follow up in years (SD)	5.82 (6.4)	13.69 (8.7)	7.29 (3.7)
Number of melanoma-specific deaths (%)	800 (13.9)	241 (4.6)	370 (19.0)

*MIA* Melanoma Institute Australia cohort, *UKB* UK Biobank, *LMC* Leeds Melanoma Cohort, *SD *Standard deviation, *N *Number, %percent

With the exception of the self-reported 23andMe, Inc. dataset, all CM cases were histopathologically confirmed; previous work has shown that 23andMe cases are very similar to the confirmed cases: the susceptibility loci show very similar effects in the self-reported and confirmed CM cases [10]. Each study was approved by the human research ethics committee at their respective institution, and all participants provided written informed consent. Specifically, for 23andMe, participants provided written informed consent and participated in the research online, under a protocol approved by the external AAHRPP-accredited IRB, Ethical & Independent Review Services.

Only SNPs with an imputation quality score > 0.5 were included, and a fixed-effects inverse variance weighted meta-analysis of log odds ratios (ORs) was performed using PLINK 1.9 [28]. Next, we selected 6,342,711 non-ambiguous, autosomal, bi-allelic GWAS meta-analysis SNPs with a MAF > 1% that were present in the validation (QSkin) and target (MIA and UKB) cohorts, and in the LD reference panel.

#### CM PRS validation cohort: The QSkin Sun and Health Study cohort

The QSkin Sun and Health Study (QSkin) cohort is a population-based cohort comprising over 43,000 adult participants recruited from Queensland, Australia. Detailed information on participant recruitment, phenotype measurement, genotyping and quality control measures have been published elsewhere [10, 33]. In summary, 18,087 participants were genotyped using the Global Screening Array [Illumina, San Diego, USA], and individuals were removed if they had non-European ancestry (6 s.d from the mean of PC1 and PC2 of 1000 Genomes European samples), were related to another participant (one from each pair removed if identity by descent pihat value > 0.1875), or had high genotype missingness (> 3%). SNPs were also removed due to HWE violations (P < 1 × 10^–6^), a low GenTrain score (< 0.6), or a low MAF (< 0.01). Cleaned genotype data were imputed to the haplotype reference consortium (v1) panel using the University of Michigan imputation server [22].

The Human Research Ethics Committee of QIMR Berghofer Medical Research Institute, Brisbane, Australia approved the study protocol and all participants provided written informed consent. We selected 16,708 participants (1,285 histopathologically confirmed CM cases and 15,423 controls) of European ancestry. CM cases were ascertained through data linkage with the Queensland Cancer Registry as well as assessing histopathology reports from pathology laboratories in Queensland.

#### Generation of the cutaneous melanoma polygenic risk score models

We used the CM-susceptibility GWAS data (generated above) and an LD reference panel of 2,000 unrelated individuals of European ancestry from UKB, to generate 30 PRS_susceptibility models at 1 megabase (Mb), 2 Mb, 3 Mb, 4 Mb and 5 Mb of LD radii each with varying fractions of causal SNPs i.e. 1 (F0), 0.1 (F1), 0.01 (F2), 0.001 (F3), 0.0001 (F4), and 0.00002 (F5). For this analysis we used LDpred, a Bayesian method that utilises all SNPs in the discovery GWAS (here CM-susceptibility GWAS), and their LD information, to derive LD-adjusted effect estimates (log ORs) for the trait (here CM-susceptibility) [34].

#### Validation of the cutaneous melanoma polygenic risk score in QSkin cohort

**Fig. 1:**
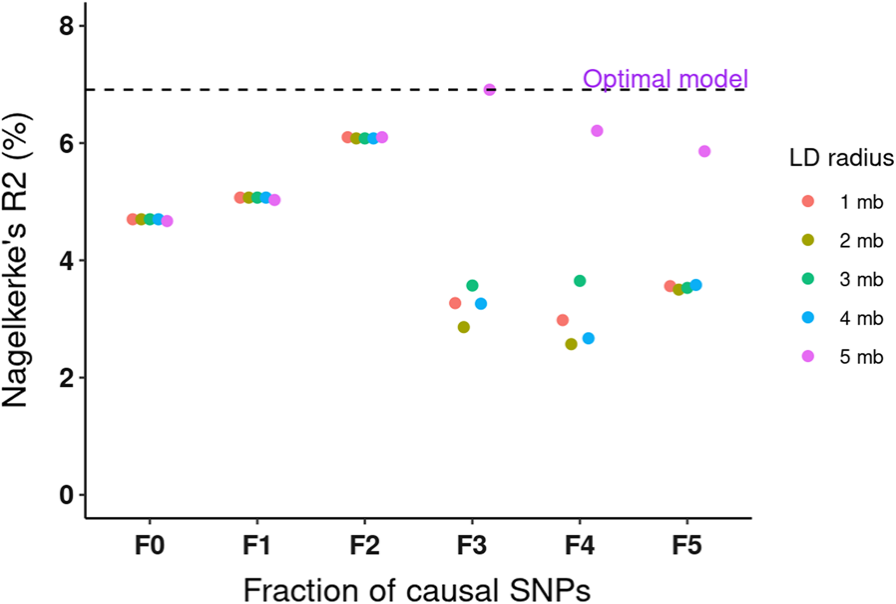
Cutaneous melanoma polygenic risk score model performance in the validation cohort (QSkin). The x-axis represents the different melanoma polygenic risk score (PRS) modelling varying fractions of causal SNPs, 1 (F0), 0.1 (F1), 0.01 (F2), 0.001 (F3), 0.0001 (F4) and 0.00002 (F5), and differing linkage disequilibrium (LD) radii, 1 megabase (Mb), 2 Mb, 3 Mb, 4 Mb and 5 Mb. The y-axis represents Nagelkerke's R^2^ (%) for each of the 30 PRS models. The horizontal dashed black line highlights the optimal model (F3 and 5 Mb) with the highest Nagelkerke’s R^2^ of 7.03%

Next, we used the QSkin validation cohort to select the optimally performing PRS. Next, for each model we computed scores for 16,708 individuals (1,285 melanoma cases and 15,423 controls) in the QSkin Cohort using the LDpred-adjusted effect sizes (log ORs) and the imputed allelic dosages using PLINK 1.9 [28]. Then we computed and used Nagelkerke’s R^2^ [35] to select the optimally performing PRS_susceptibility model by comparing the model fit for CM risk ~ PRS_susceptibility + age + sex + 10 PCs, and a null model (CM risk ~ age + sex + 10 PCs) using the PredictABEL R package [36]. Model performances are presented in Fig. 1, and the best performing PRS model was used in the subsequent analyses.

#### Testing for association between cutaneous melanoma polygenic risk scores and melanoma-specific survival

The best performing PRS_susceptibility model was applied to the MIA and UKB cohorts using imputed allelic dosages and PLINK 1.9. The PRS was normalised to have a mean of 0 and an SD of 1 and tested for association with MSS in a Cox proportional hazard model adjusted for age at diagnosis, sex and the first ten PCs using the *survival* package in R [29]. We further calculated the MSS HR and 95% confidence interval (CI) per SD increase in the PRS_susceptibility. Next, we conducted a fixed- and random- effects inverse-variance meta-analysis to compute the pooled HR and 95% CI using the meta R package [37]. We then tested for association between MSS and the same PRS_susceptibility in the LMC, adjusting for the same covariates. Finally, we meta-analysed the MIA, UKB and LMC results.

#### Sensitivity analyses for polygenic susceptibility to melanoma and melanoma-specific survival

Pigmentation and naevus count loci are major biological pathways for CM-susceptibility [10, 38]. We further explored whether any association between the PRS_susceptibility and MSS was driven by SNPs associated with pigmentation and/or naevi pathways (Additional file 1). In addition, we generated PRSs for pigmentation (PRS_P_), naevus count (PRS_N_) and telomere length (PRS_TL_) and tested whether they were associated with MSS (Additional file 1). To rule out the possibility of thin or slow-growing melanomas influencing the PRS-survival association, we explored the potential influence of tumour stage, thickness and lead-time bias on any associations (Additional file 1). To test, and adjust, for the presence of an index-event bias we used the method of Dudbridge et al., 2019 [39] as implemented in the indexevent R package (https://rdrr.io/github/DudbridgeLab/indexevent/man/indexevent.html). The MIA survival GWAS, CM risk GWAS meta-analysis, and LD panel (5000 random individuals from the UK Biobank) were filtered to a common set of SNPs, and then pruned to LD independence (LD r^2^ < 0.01) in plink v1.9b6.26, leaving 102,286 SNPs. As per Dudbridge et al., 2019, the log(HR) effect sizes from the MIA and UKB survival GWAS were regressed onto the log(OR) effect sizes from the CM risk GWAS meta-analysis, and SCIMEX (1000 simulations) was used to adjust the regression slope for regression dilution to calculate the correction factor, and its variance, which was used to adjust the survival GWAS results. The impact of index-bias on the association between polygenic risk for melanoma, and survival was estimated as done in Howe et al. [40]. Briefly, using the LD pruned set used to calculate the correction factor above, 57 SNPs with a CM risk P < 5 x 10^-8^ and independent (LD r^2 ^<  0.01) were selected, and the inverse-variance weighted method (implemented in the R MendelianRandomization package [41]) was used to estimate the association between polygenic risk for melanoma and survival before and after the correction.

## Results

### Baseline characteristics of the melanoma survival cohorts

This analysis was restricted to 5,762 melanoma patients in the MIA cohort, 5,220 in the UKB cohort, and 1,947 in the LMC. Summary data on mean age at diagnosis, sex, duration of follow up and the number of melanoma-specific deaths are presented in Table 1.

### Genome-wide significant genetic variants for melanoma-specific survival

A MSS GWAS meta-analysis of the MIA and UKB cohorts identified two independent genome-wide significant (P < 5 × 10^–8^) loci (Table 2, and Additional file 1: Figure S1); rs41309643 (P = 2.08 × 10^–8^) on chromosome 1 (1q42.13) and rs75682113 (P = 1.07 × 10^–8^) on chromosome 7 (7p14.1) (Table 2). However, neither SNP was replicated in the LMC (rs41309643 P = 0.679 and rs75682113 P = 0.411 (Table 2, and Additional file 2: Table S2). Following the meta-analysis of all three cohorts, rs41309643 was no longer formally significant at P < 5 × 10^–8^ (HR = 1.83, 95% CI = 1.45–2.30, P = 3.21 × 10^–7^) with high heterogeneity metrics (Table 2). rs75682113 remained genome-wide significant with no significant evidence of heterogeneity (C-allele HR = 2.23, 95% CI = 1.68–2.95, P = 2.13 × 10^–8^; Table 2).

**Table 2:** Genetic variants for melanoma-specific survival in the discovery cohorts (MIA + UKB) and replication cohort (LMC)

						Meta-analysis of MIA and UKB							Meta-analysis of MIA, UKB and LMC						
						Fixed effects		Random effects		Heterogeneity			Fixed effects		Random effects		Heterogeneity		
SNP	CHR	BP	Gene*	EA/ NEA	EAF	HR (95% CI)	P	HR (95% CI)	P	Direction	I^2^	Q	HR (95% CI)	P	HR (95% CI)	P	Direction	I^2^	Q
rs41309643	1	227,078,509	*COQ8A/PSEN2*	G/T	0.018	2.09 (1.61–2.71)	2.81 × 10^–8^	1.89 (1.12–3.19)	0.0179	p + +	64.9	0.09	1.83 (1.45–2.30)	3.24 × 10^–7^	1.58 (0.95–2.62)	0.078	p + + +	74.1	0.02
rs75682113	7	40,708,001	*SUGCT*	C/G	0.020	2.38 (1.77–3.21)	1.07 × 10^–8^	2.38 (1.77–3.21)	1.07 × 10^–8^	p + +	0.0	0.39	2.23 (1.68–2.95)	2.14 × 10^–8^	2.17 (1.57–3.02)	3.45 × 10^–6^	p + + +	19.6	0.29

*MIA* Melanoma Institute Australia cohort, *UKB* UK Biobank cohort, *LMC* Leeds Melanoma Cohort, *SNP* single nucleotide polymorphism, *CHR* chromosome, *BP* Hg19 base position, *EA* effect allele, *NEA* non-effect allele, *EAF* effect allele frequency reported from the Haplotype Reference Consortium (HRC), *HR* hazard ratio, *CI* confidence interval, *P* P-value. Gene* rs41309643 is an eQTL for the C0Q8A gene in blood (eQTL P = 9.3 × 10^–14^), as well as closest to and an eQTL for the PSEN2 gene (eQTL P = 5.5 × 10^–5^) in GTEx/v8

Rs41309643 on chromosome 1 is an intron of the *PSEN2* gene and is associated with the expression of the *Coenzyme Q8A* (*COQ8A*) (formerly *ADCK3*) gene in blood. *COQ8A* is induced by *p53* in response to DNA damage and inhibition of *COQ8A* counteracts p53-induced apoptosis [42]. rs75682113 on chromosome 7 is in an intron of the *Succinyl-Coa:Glutarate-Coa Transferase* (*SUGCT*) gene. This SNP has not been reported as an *eQTL* for any genes. Independent variants in the *SUGCT* gene have been associated with glutaric aciduria type 3 disease susceptibility [43].

### The optimal cutaneous melanoma susceptibility polygenic risk score model

Of the thirty PRS tested, the model with the F3 causal fraction (0.001) and a 5 Mb LD radius performed best, with a Nagelkerke’s R^2^ of 7.02% (Fig. 1), and was used in all subsequent analyses.

### Association of polygenic susceptibility to melanoma and melanoma-specific survival

**Fig. 2:**
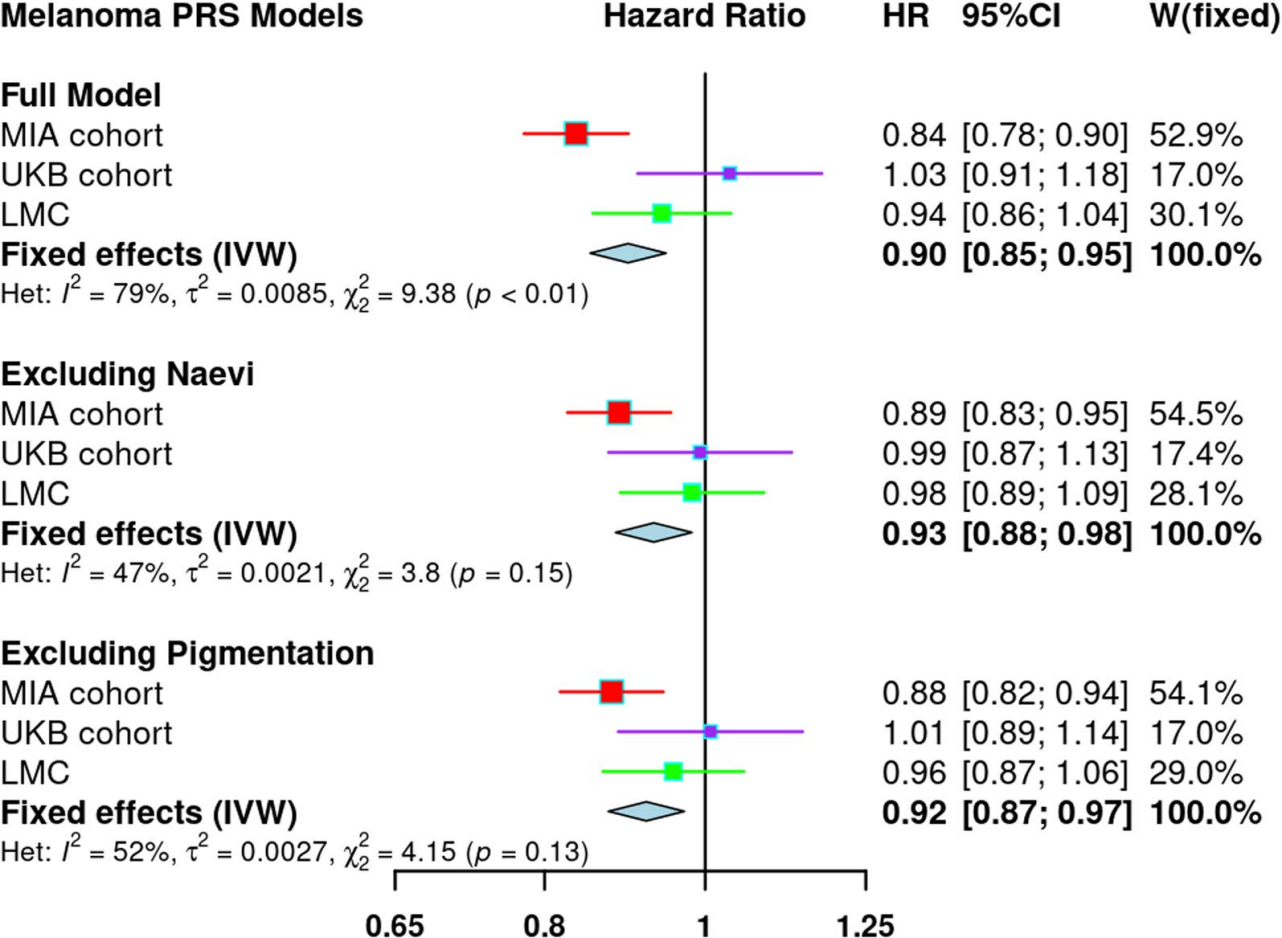
Association of polygenic risk for cutaneous melanoma and melanoma-specific survival. All models were adjusted for age, sex and the first 10 PCs and additionally genotype batch effects in the MIA analysis. HRs were estimated using Cox proportional-hazards models. The full model refers to the PRS_susceptibility (CM PRS), while for the remaining two models the PRS_susceptibility respectively excluded SNPs in the naevus count and pigmentation pathways. MIA- Melanoma Institute Australia, UKB—United Kingdom Biobank, LMC—Leeds Melanoma Cohort, IVW- Inverse variance weighted methods, Het- heterogeneity, HR- hazard ratio. CI- confidence interval

After adjusting for age at diagnosis, sex and the first ten PCs, a one SD increase in the PRS_susceptibility was associated with improved MSS in a fixed-effects meta-analysis of MIA and UKB cohorts (HR = 0.88, 95% CI = 0.83–0.94, P = 6.93 × 10^–5^). However, the association between the PRS_susceptibility and MSS was highly heterogeneous across the two studies (I^2^ = 87.7%, 95% CI = 52.4–96.8%), with the finding mainly driven by the high UV setting cohort (MIA HR = 0.84, 95% CI = 0.78–0.90). Although not statistically significant, the magnitude and direction for the random effects model was also consistent with the fixed-effects results (fixed effects model HR = 0.92, 95% CI = 0.75–1.13, P = 0.43). The inverse association between polygenic susceptibility to melanoma and MSS persisted after excluding genomic regions associated with naevus count (fixed-effects HR = 0.91, 95% CI = 0.86–0.97, P = 0.0038; random-effects HR = 0.93, 95% CI = 0.83–1.03, P = 0.16) and pigmentation (fixed-effects HR = 0.91, 95% CI = 0.850–0.97, P = 0.0023; random-effects HR = 0.93 95% CI = 0.82–1.06, P = 0.26). The association between polygenic risk for melanoma and MSS was not replicated (P > 0.05) in the LMC; however, the directions of the effect estimates were consistent (Fig. 2).

In a meta-analysis of the three cohorts a one SD increase in the PRS_susceptibility was still associated with improved MSS (fixed-effects HR = 0.90, 95% CI = 0.85–0.95, P = 6.35 × 10^–5^; random-effects HR = 0.93, 95% CI = 0.83–1.04, P = 0.20), even after excluding naevus and pigmentation loci (Fig. 2). There was substantial heterogeneity across the three studies (I^2^ = 78.7%, 95% CI = 31.6–93.4%). Sensitivity analyses showed that the skin colour PRS was also associated with improved MSS (PRS_P_; HR = 0.90, 95% CI = 0.85–0.96, P = 1.1 × 10^–3^), while the naevus count PRS also provided suggestive evidence (PRS_N_; HR = 0.95, 95%CI = 0.89–1.02, P = 0.179) (Additional file 1: Figure S5).

### Influence from melanoma prognostic factors, lead-time, and index-event biases in the MIA Cohort

**Fig. 3:**
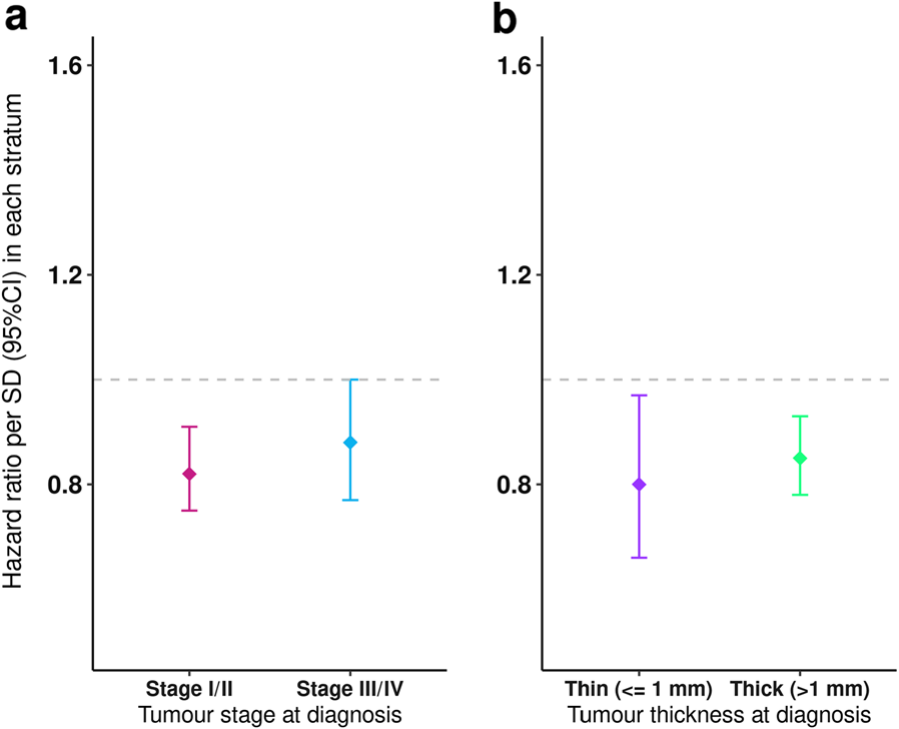
Stratified analysis of the PRS and MSS association by the AAJC Stage and primary tumour thickness in the MIA Cohort. The y-axis represents the hazard ratio for MSS per standard deviation (SD) increase in the PRS. Error bars show the 95% confidence interval of the HR. The x-axis shows the strata for tumour stage and thickness at diagnosis of melanoma. The dashed grey line represents a null effect at a hazard ratio of 1. **Panel 3a** shows the association between the CM PRS and MSS stratified by the AJCC 2010 tumour stage, after controlling for age at diagnosis, sex, the first 10 ancestral components and genotype batch effects. Stage I/II included 4493 participants and 427 melanoma deaths, while stage III/IV included 789 participants and 242 melanoma deaths. **Panel 3b** reports the association of the CM PRS and MSS stratified by the primary tumour thickness, after controlling for age at diagnosis, sex, the first 10 ancestral components and genotype batch effects. The thin (< = 1 mm) stratum included 1,898 participants and 122 melanoma deaths, while the thick (> 1 mm) stratum included 3,384 participants and 547 melanoma deaths

In the MIA cohort the PRS_susceptibility remained associated with improved survival after excluding participants with melanoma *in-situ*, and those with an unknown stage (HR = 0.84, 95% CI = 0.78–0.90, P = 2.15 × 10^–6^). In addition, the association was consistent even after adjusting for age, sex, 10 PCs, AJCC 2010 Stage, and primary tumour thickness (HR = 0.84, 95% CI = 0.78—0.91, P = 1.90 × 10^–5^) (Table 3). There was also no evidence for interaction by the tumour stage or tumour thickness (Table 3). In a stratified analysis, there was no evidence that the association between the PRS and MSS differed by tumour stage (Fig. 3a) and primary tumour thickness at diagnosis (Fig. 3b). The PRS_TL_ was suggestive but not significantly associated with MSS in the MIA cohort (PRS_TL_; HR = 0.90, 95% CI = 0.64–1.27, P = 0.5504). After excluding the first two years of follow-up (following diagnosis), there was no evidence of lead-time bias (survival bias) (HR = 0.84, 95% CI = 0.77–0.91, P = 4.03 × 10^–5^). We applied the index-event bias proposed in Dudbridge et al. [39] to the MIA survival GWAS results; the results for both individual SNPs, and estimates of the association of polygenic risk and survival, were essentially unchanged before and after the correction [data not shown].

**Table 3:** Testing for an interaction between the polygenic susceptibility to melanoma and survival prognostic factors in the MIA Cohort

Model	N	Events	HR	95% CI	P-value
PRS + Age + Sex + Breslow + Stage + 10PCs + Batch	5282	669	0.84	0.78–0.91	1.9 × 10^–5^
PRS*Stage + Age + Sex + Breslow + 10PCs + Batch	5282	669	0.82	0.75–0.91	1.6 × 10^–4^
PRS*Breslow + Stage + Age + Sex + 10PCs + Batch	5282	669	0.84	0.70–1.01	0.060
PRS*Breslow*Stage + Age + Sex + 10PCs + Batch	5282	669	0.85	0.70–1.03	0.095

*N* number of participants, *HR* hazard ratio, *CI* confidence intervals

## Discussion

In this study, we performed the largest GWAS for MSS to date using data from Australia and the United Kingdom and potentially have identified two independent, novel, genome-wide significant (P < 5 × 10^–8^) loci for MSS at 1q42.13 and 7p14.1. While the two loci did not formally replicate in an independent cohort, the confidence intervals (particularly for rs75682113) in the replication set overlap the estimate from the discovery cohorts. Confirmation of these two loci will require replication in larger cohorts. rs75682113 is particularly promising as it was genome-wide significant (P < 5 × 10^–8^) in our meta-analysis of the discovery and replication samples.

In addition, we report evidence that increased genetic susceptibility for CM, as measured by a one SD increase in a PRS_susceptibility, was significantly associated with improved MSS. However, caution is required as the result was primarily driven by a strong association in the MIA cohort. Genetic susceptibility to CM is primarily driven by loci in the pigmentation and naevus count pathways [44]. HRs for PRS_susceptibility and MSS were slightly attenuated (but still with a significant association) when we removed SNPs in either pathway. In turn PRS designed specifically for these traits were also associated (though not significantly for naevus count) with MSS. In addition, the PRS for telomere length (another pathway to both CM susceptibility and survival) was not significantly associated with MSS in our sensitivity analysis. These pathway-analysis results suggest that if genetic propensity to CM is associated with improved survival it is not simply due to pigmentation, nevus count or telomere length.

However, this study suggests that if there is a true association, its magnitude may differ across populations, presumably due to environmental factors such as high UV (e.g. in Australia) and other effects. Firstly, the MIA and UKB meta-analysis results did not replicate in the LMC. Secondly, the high heterogeneity metrics (e.g. I^2^) indicate that the effect sizes may not be consistent across the three studies, with a very strong result in the MIA cohort (a high UV setting) and weaker associations in the UK samples (Table 1 and Additional file 2: Table S2). Although the fixed-effects model shows a strong statistically significant association, the results are not significant for the random-effects model even when they are of a similar magnitude. The observed heterogeneity may be due to differences in recruitment, where the MIA cohort recruitment was from clinics as opposed to the population-based UKB and LMC. It is also possible that the strong inverse result in Australia is influenced by overdiagnosis for melanoma [45]. It is estimated that 54% of all melanomas and 15% invasive melanomas in Australia are over-diagnosed [46]. Thus, patients may be diagnosed with non-lethal melanoma and subsequently exhibit improved survival. However, recent evidence suggests that regular skin checks (which may lead to overdiagnosis for melanoma) are not associated with MSS [47]. Since sun exposure or high UV exposure is associated with improved MSS [48, 49], it is also possible that differences in high or long-term sun and ultraviolet-radiation exposure in Australia are in part responsible for the heterogeneity.

Analyses of outcomes such as survival necessitate the inclusion of people based on having the index disease, which can introduce an index event bias [39]. This can potentially lead to spurious associations between disease risk factors (such as SNPs associated with risk of disease) with survival. The application of a recent method to identify and adjust for this bias in our data did not meaningfully change the results, similar to what has been observed for previous studies [40].

In a more detailed analysis in the MIA cohort, our study suggests that this inverse association is consistent even after further adjusting for (and testing for interaction with) strong predictors of MSS like tumour stage and primary tumour thickness at diagnosis. The stratified analysis shows that the association is not modified by primary tumour thickness or stage. Thus, if replicated in additional cohorts, a CM-susceptibility PRS is potentially an independent prognostic factor for MSS.

To our knowledge, while no prior study has examined the association of a CM susceptibility PRS and survival outcome, similar inverse relationships have been found in other cancers e.g. higher breast cancer PRSs and better breast cancer prognosis/characteristics [50, 51]. Also, a follicular lymphoma PRS was associated with improved overall survival among women in a population in the USA [52]. BRCA1/2 mutations which increase breast cancer risk were associated with better overall survival among triple-negative breast cancer women [53]. A CAD PRS was inversely associated with all-cause mortality (OR = 0.91; 95% CI = 0.85–0.98), and ischaemic stroke (OR = 0.78; 95% CI = 0.67–0.90) in CAD patients [40].

The mechanisms underlying this inverse association are unclear. Particularly for MSS, it could be that a higher genetic risk for CM leads to thin melanomas or slow-growing melanomas that are less lethal [54, 55, 56], and respond better to treatment. However, detailed analysis in the MIA cohort showed no difference in survival for both thin and thick tumour categories. In addition, after excluding the initial two years of follow up, the results were consistent, suggesting there is no survival/ lead-time bias.

As noted in our study above, higher nevus counts may be associated with a lower chance of dying from melanoma [14]. It is possible however that those with large numbers of naevi are subjected to increased screening, which may lead to overdiagnosis and greater survival relative to those with fewer moles [57]. However, as already indicated, increased screening is not associated with MSS [47].

Another possible mechanism could be via gene-environment interaction, where those at highest genetic risk of CM benefit more from treatment (e.g. immunotherapy), as it is the case for those at high genetic risk for coronary artery disease (CAD) and treatment benefits from PCSK9 inhibitors in the FOURIER and ODYSSEY OUTCOMES trials [58, 59].

This study presents new insights that highlight the potential clinical utility of *PRS_susceptibility* for profiling and monitoring patients for melanoma outcomes following diagnosis during the “melanoma follow-up care program” [60, 61]. In combination with other prognostic factors, it could be used to guide patient care e.g. counselling on modification of mortality-related non-genetic behaviours and lifestyle factors, or guide the direction of patient-specific treatment to help improve survival after diagnosis. It may also be useful for the stratification of patients while recruiting into clinical trials evaluating melanoma treatment and outcomes.

## Conclusions

In a GWAS meta-analysis of MSS, we identified two novel loci potentially associated with survival from cutaneous melanoma, both of which contain candidate genes linked to tumour progression; however, replication in large independent cohorts is required. In line with observations in other cancers and complex diseases, increased germline genetic susceptibility for CM was strongly but heterogeneously associated with improved MSS especially in a high UV setting. If validated, a PRS_susceptibility could be used to predict melanoma outcomes after diagnosis and profile patients for personalised care.

## Supplementary Information

**Additional file 1: Figure 1**. Manhattan plot for the MSS GWAS meta-analysis between MIA and UKB cohorts. **Figure 2**. Skin colour polygenic risk score model performance in the validation cohort (QSkin). The x-axis represents the different melanoma polygenic risk score (PRS) models of varying fractions of causal SNPs (i.e. 1 (F0), 0.1 (F1), 0.01 (F2),0.001(F3),0.0001 (F4) and 0.00002 (F5)) at the different radii of the linkage disequilibrium (LD) (i.e. 2 megabase (mb) and 5 mb). The y-axis represents Nagelkerke’s R2 (%) for each of the 12 PRS models. The horizontal dashed black line highlights the optimal model (F1-2mb) (i.e. with the highest Nagelkerke’s R2). **Figure 3**. The association between polygenic risk scores and the risk of melanoma in QSkin. **Figure 4**. The association by quartile of polygenic risk for melanoma susceptibility and melanoma specific survival in the MIA cohort. **Figure 5**. Association of standalone skin colour and naevus PRSs and melanoma specific survival in MIA and UKB.

**Additional file 2: Table 1**. Cohorts used for the cutaneous melanoma susceptibility discovery meta-analysis GWAS. **Table 2**. Per study melanoma specific survival  GWAS results. **Table 3**. Pigmentation loci excluded from the cutaneous melanoma susceptibility polygenic risk score. **Table 4**. Naevi loci excluded from the cutaneous melanoma susceptibility polygenic risk score. **Table 5**. Naevous count SNPs included in the naevus polygenic risk score.

## Data Availability

CM GWAS summary statistics used to generate the LDPred PRSs can be accessed as indicated by Landi et al. 2020. Underlying data for the cohorts used in the paper are available through application to the respective cohorts; UKB (http://www.ukbiobank.ac.uk/wp-content/uploads/2012/09/Access-Procedures-2011-1.pdf); MIA (https://www.melanoma.org.au/research/collaborate-on-research-with-mia/); Q-Skin (By application to Q-Skin Principal Investigator David Whiteman David.Whiteman@qimrberghofer.edu.au).

## Abbreviations

AJCC: American Joint Committee on Cancer
CM: Cutaneous melanoma
GWAS: Genome-wide association studies
HR: Hazard ratio
ICD: International Classification of Diseases
Mb: Megabase
LD: Linkage disequilibrium
MIA: Melanoma Institute Australia
MSS: Melanoma-specific survival
MAF: Minor allele frequency
PRS: Polygenic risk score
PCs: Principal components
QSkin: QSkin Sun and Health Study
SNP: Single nucleotide polymorphism
SD: Standard deviation
UKB: UK Biobank
R^2^: Variance
LMC: Leeds Melanoma Cohort
CI: Confidence interval (CI)
